# Sensors for Highly Toxic Gases: Methylamine and Hydrogen Chloride Detection at Low Concentrations in an Ionic Liquid on Pt Screen Printed Electrodes

**DOI:** 10.3390/s151026866

**Published:** 2015-10-22

**Authors:** Krishnan Murugappan, Debbie S. Silvester

**Affiliations:** Nanochemistry Research Institute, Department of Chemistry, Curtin University, GPO Box U1987, Perth 6845, WA, Australia; E-Mail: d.silvester-dean@curtin.edu.au

**Keywords:** room temperature ionic liquids, screen printed electrodes, cyclic voltammetry, differential pulse voltammetry, square wave voltammetry, detection limit, gas sensing

## Abstract

Commercially available Pt screen printed electrodes (SPEs) have been employed as possible electrode materials for methylamine (MA) and hydrogen chloride (HCl) gas detection. The room temperature ionic liquid (RTIL) 1-ethyl-3-methylimidazolium bis(trifluoromethylsulfonyl)imide ([C_2_mim][NTf_2_]) was used as a solvent and the electrochemical behaviour of both gases was first examined using cyclic voltammetry. The reaction mechanism appears to be the same on Pt SPEs as on Pt microelectrodes. Furthermore, the analytical utility was studied to understand the behaviour of these highly toxic gases at low concentrations on SPEs, with calibration graphs obtained from 10 to 80 ppm. Three different electrochemical techniques were employed: linear sweep voltammetry (LSV), differential pulse voltammetry (DPV) and square wave voltammetry (SWV), with no significant differences in the limits of detection (LODs) between the techniques (LODs were between 1.4 to 3.6 ppm for all three techniques for both gases). The LODs achieved on Pt SPEs were lower than the current Occupational Safety and Health Administration Permissible Exposure Limit (OSHA PEL) limits of the two gases (5 ppm for HCl and 10 ppm for MA), suggesting that Pt SPEs can successfully be combined with RTILs to be used as cheap alternatives for amperometric gas sensing in applications where these toxic gases may be released.

## 1. Introduction

Miniaturization has been the driving force for many industries and it is likely that the electrochemical sensor industry is not far behind. Planar devices (e.g., screen printed electrodes, or SPEs) where all three electrodes are contained within a very small area have become increasingly more popular for use as electrode materials in amperometric gas sensors (AGSs) [1,2,3,4,5,6], but they have yet to become commercialised (in AGSs). SPEs contain various materials (e.g., graphite, carbon black and polymeric binders) that are squeezed through a mesh screen which defines the shape and size of the electrode [7]. They are relatively cheap and portable, and can be combined with non-volatile and highly thermally stable room temperature ionic liquid (RTIL) solvents to make more robust sensors. Previous work on ammonia [1], chlorine [8] and oxygen [2] gas-sensing on SPEs has already been reported and shows reasonable limits of detection (LODs), but not as low as the exposure limits for ammonia and chlorine, since lower concentration ranges were not studied. The real question is: can RTIL solvents be used with screen printed electrodes in amperometric gas sensors to detect analyte gases at levels at or below the exposure limits?

Many studies on gases in RTILs have so far focussed on higher concentrations with only one group to report ammonia gas-sensing in the ppm range (0 to 40 ppm), with LODs between 0.2 and 2 ppm in a range of RTILs [9]. The two gases that are the focus of this work (MA and HCl) are highly toxic at low ppm concentrations. For example, methylamine is a colourless and toxic gas with a distinct fish-like odour at concentrations above 100 ppm [10]. The current (USA) Occupational Safety and Health Administration Permissible Exposure Limit (OSHA PEL) is 10 ppm in the gas phase. HCl is highly corrosive and, in the presence of moisture, forms hydrochloric acid which causes burns and irritation to the skin, as well as corrosion to metals (e.g., iron) [11]. The current (USA) OSHA PEL of HCl is 5 ppm in the gas phase. Due to their high toxicity, it is therefore essential to be able to monitor and detect methylamine and hydrogen chloride gases at low ppm concentrations.

Current technology to detect methylamine and hydrogen chloride gases exists, but is limited. To the best of our knowledge, there are no commercial AGSs for methylamine, but colorimetric gas detectors (Sensidyne, 1–20 ppm, operating temperature up to 40 °C) and photoionization detectors (Mil-Ram Technology, 0–200 ppm, operating temperature up to 55 °C) are available. For HCl, companies such as Membrapor and Alphasense manufacture AGSs in the range of 0–20 ppm, but the operating temperatures do not go above 45 °C. This clearly shows that RTIL-based sensors have the possibility to be used in sensors over a much wider temperature range due to their non-volatility and high thermal stability.

In previous works the electrochemical behaviours of methylamine [12] and hydrogen chloride [13] gases have been investigated in room temperature ionic liquids (RTILs) on conventional Pt micro/macrodisk electrodes. In this work, the electrode reaction mechanism will first be investigated to see if the behaviour of these toxic gases on SPEs is similar to conventional electrodes. Then various concentrations will be studied to obtain analytical parameters (LODs and sensitivities) so that a direct comparison of LODs can be made with conventional Pt microdisk electrodes in the RTIL [C_2_mim][NTf_2_]. It is important to note that, in previous work, the mechanism for reduction of oxygen on SPEs was different from that on microelectrodes in this RTIL (reaction of the reduction product with the materials in the SPE paste) [2] and indicated that the detection of oxygen on Pt SPEs may not be feasible for long-term studies. This work will help determine whether RTILs and SPEs can be used in the design of cheap, portable and robust gas sensors for toxic gases such as methylamine and hydrogen chloride.

## 2. Experimental Section

### 2.1. Chemical Reagents

First 1-ethyl-3-methylimidazolium bis(trifluoromethylsulfonyl)imide ([C_2_mim][NTf_2_]) was purchased from Merck Millipore (Victoria, Australia). Ultra-pure water with a resistance of 18.2 MΩ·cm prepared by an ultra-pure laboratory water purification system (Millipore Pty Ltd., North Ryde, NSW, Australia) and acetonitrile (MeCN, Sigma-Aldrich, 99.8%) were used for washing the electrodes after use with RTILs. Methylamine (0.2% nitrogen fill) and hydrogen chloride gas (0.2% nitrogen fill) was purchased from CAC gases (Auburn, NSW, Australia). High purity nitrogen gas (99.9%) was from BOC gases (North Ryde, NSW, Australia) and used as a carrier gas.

### 2.2. Electrochemical Experiments

All electrochemical experiments were performed using a PGSTAT101 Autolab potentiostat (Eco Chemie, Utrecht, The Netherlands) interfaced to a PC with NOVA version 1.9 software. Pt SPEs employed in this study were purchased from DropSens (DRP-550, Oviedo, Spain). The SPEs consist of a three-electrode setup with a Pt working, Pt counter and Ag reference electrode. The SPEs were housed in a glass “T-cell” previously used to study ammonia [1] and oxygen [2] gases. Before the introduction of gas, the cell was purged under nitrogen to remove any impurities present in the RTIL (volume of 10 µL). Although it is widely accepted that vacuum techniques are superior to purge the RTIL of atmospheric impurities found in RTILs [14,15,16], it was not possible to use the vacuum due to the violent degassing in the current experimental set-up (where a layer of RTIL is spread over the flat surface of the SPE, in contrast with other work [14] where the RTIL is neatly contained within the cap of a pipette tip). The SPEs were used “as is” without any activation/precleaning as this represents the conditions required in the real world.

### 2.3. Gas-Mixing Setup

A similar gas-mixing setup as used by Lee *et al.* [2] was employed to obtain different concentrations of methylamine and hydrogen chloride. The % (or ppm) concentration of methylamine or hydrogen chloride that was introduced into the T-cell was calculated using the relative flow rates of the two flow meters. A digital flowmeter (0–1.2 L/min, John Morris Scientific, NSW, Australia) was used for the nitrogen gas and an analogue flowmeter (0–60 cm^3^/min, Dwyer, NSW, Australia) was used for hydrogen chloride/methylamine gas. Approximately 30 min was allowed for saturation to occur for each specific concentration and a stable response was determined when the current was identical in two cyclic voltammograms (CVs) taken 10 min apart. Response times were not investigated.

## 3. Results and Discussion

All experiments were conducted with the RTIL [C_2_mim][NTf_2_], which has a high intrinsic conductivity (8.8 mS·cm^−1^) and is one of the least viscous RTILs available (34 cP at 293 K) [17], suggesting a faster gas saturation time. It has also been observed previously (on a platinum microelectrode) that a linear current response *vs.* concentration can be obtained in [C_2_mim][NTf_2_] for one of the gases under study (methylamine [12]). We are currently performing mechanistic studies for HCl on a Pt microelectrode in a range of RTILs, which will be the focus of a future publication.

### 3.1. Methylamine Gas

#### 3.1.1. Electrochemical Oxidation of Methylamine Gas on Pt SPEs

**Figure 1:**
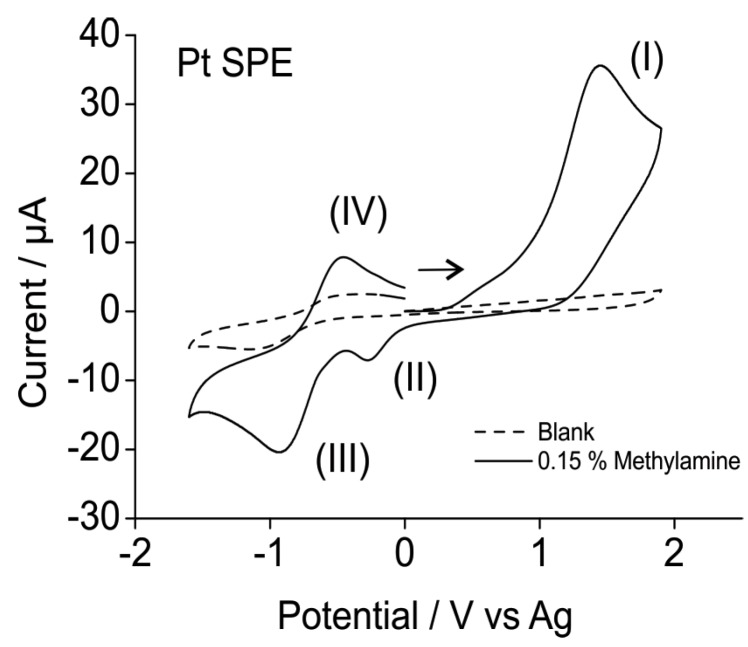
CV for the oxidation of 0.15% methylamine gas on a Pt SPE in [C_2_mim][NTf_2_] at a scan rate of 0.1 V/s. Dotted line is the response in the absence of methylamine.

Figure 1 shows the electrochemical oxidation of 0.15% methylamine gas on a platinum SPE in the RTIL [C_2_mim][NTf_2_]. The mechanism for the oxidation reaction has been elucidated in our previous work [12] and therefore will not be discussed in detail here. In brief, methylamine is oxidised (at *ca.* 1.5 V) to form ammonia which itself undergoes oxidation at that potential. The combination of these two processes produces the oxidation peak (labelled peak I on Figure 1), which will be used as the analytical response. The oxidation peak (I) is followed by two reduction peaks (II and III) at −0.2 V and −0.9 V and a subsequent oxidation peak (IV) at −0.4 V. These processes correspond to: (II) the reduction of solvated protons, (III) the reduction of ammonium ion and (IV) the oxidation of adsorbed hydrogen.

The blank scan (RTIL in the absence of methylamine, dotted line, Figure 1) also shows an electroactive reduction wave at *ca.* −1.2 V *vs.* Ag, which is believed to correspond to an impurity in the RTIL, possibly left over from the synthesis (e.g., unreacted imidazole). This does not affect the current for methylamine oxidation, since peak (I) occurs at much higher potentials (*ca.* 1.5 V), where the background current is low.

**Figure 2:**
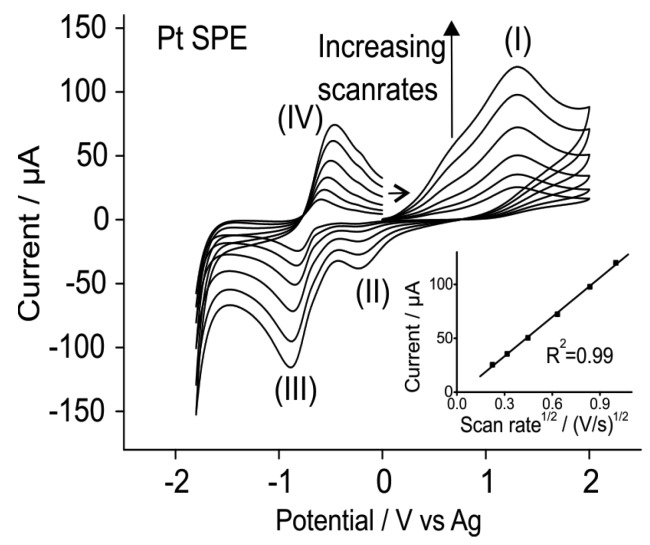
CV for the oxidation of 0.15% methylamine gas on a Pt SPE in [C_2_mim][NTf_2_] at varying scan rates between 0.05 and 1 V/s. The inset shows the plot of peak current *vs.* the square root of scan rate for peak (I).

Figure 2 shows cyclic voltammetry for the oxidation of 0.15% methylamine at varying scan rates from 0.05 to 1 V/s when scanned from 0 to 2 V and back to −1.8 V. It can be seen that as the scan rates increase, the peak currents for all four peaks increase. The inset of Figure 2 shows a plot of the peak current for the oxidation peak (I) *vs.* the square root of the scan rate. A linear response is obtained, suggesting that the process occurring is diffusion-controlled, consistent with that observed previously on a Pt microelectrode [12]. These results are encouraging as it shows that the behaviour on a Pt SPE is very similar to a Pt microelectrode. It has been shown previously that oxygen voltammetry on SPEs in imidazolium RTILs [2] is not ideal, since the voltammetry was very different on a SPE compared to a microelectrode, suggesting that SPEs may not be suitable for oxygen gas detection in these RTILs. In the present case, it has been established that the behaviour on a SPE is similar to that on a microelectrode, so a study of the current response *vs.* concentration can be carried out for methylamine gas on SPEs.

#### 3.1.2. Analytical Utility of Methylamine Gas on Pt SPEs

**Figure 3:**
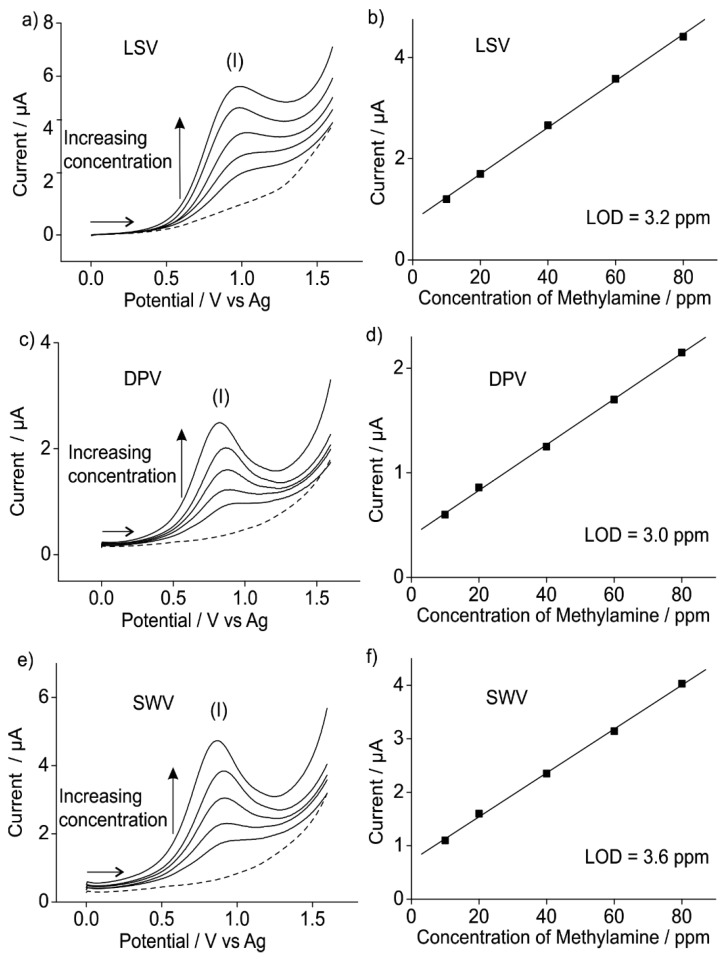
(**a**) LSV, (**c**) DPV and (**e**) SWV for the oxidation of methylamine gas at different concentrations (10, 20, 40, 60 and 80 ppm in the gas phase) on a Pt SPE in [C_2_mim][NTf_2_]. Dashed line is the response in the absence of methylamine gas. The corresponding calibration plots obtained for (**b**) LSV, (**d**) DPV and (**f**) SWV using the peak currents from (**a**,**c**,**e**) are also shown.

To study the analytical response, a lower concentration range for methylamine gas was employed and three different techniques were explored: linear sweep voltammetry (LSV), differential pulse voltammetry (DPV) and square wave voltammetry (SWV). DPV and SWV are often employed as more sensitive techniques compared to cyclic voltammetry/linear sweep voltammetry due to the lower impact of the charging current [18]. Figure 3 shows the oxidation of methylamine gas at five different concentrations (from 10 to 80 ppm) on a Pt SPE in [C_2_mim][NTf_2_] at a scan rate of 0.1 V/s using LSV, DPV and SWV for the oxidation peak (I). The optimised parameters for DPV were an amplitude of 0.05 V, modulation time of 0.025 s and interval time of 0.05 s. For SWV, the optimised parameters were an amplitude of 0.05 V and frequency of 20 Hz. It can be seen that as the concentration of methylamine increases, the current for all the oxidation peaks increases. The peak current was plotted against the respective concentrations to obtain calibration graphs, as shown in Figure 3b,d,f. Excellent linearity (*R*^2^ > 0.99) was obtained for the concentration range studied. LODs of 3.2, 3.0 and 3.6 ppm, and sensitivities of 4.6 × 10^−8^, 2.2 × 10^−8^ and 4.1 × 10^−8^ A/ppm were obtained for LSV, DPV and SWV, respectively. The LODs were calculated using three times the standard deviation (3σ) of the slope of the calibration line. The LODs are much better than those obtained on a Pt microelectrode (15.7, 14.2 and 15.9 ppm for LSV, DPV and SWV, respectively; these results are shown in Figure S1 of the supporting information). These results are highly encouraging and show that Pt SPEs can be used for methylamine gas detection at low ppm levels, allowing for cheaper and more miniaturised AGSs for methylamine. There has only been one report of LODs under 10 ppm for gases in pure RTILs, which was for ammonia gas on Pt printed electrodes [9], but this is the first time such low LODs for methylamine gas have been reported. The LOD is less than the OSHA PEL limit for methylamine (10 ppm), suggesting that RTIL/SPE-based sensors have the potential to be used in commercial sensors where humans might be exposed to methylamine.

### 3.2. Hydrogen Chloride

#### 3.2.1. Electrochemical Oxidation of Hydrogen Chloride Gas on Pt SPEs

The electrochemical reaction mechanism for HCl gas has been elucidated previously on a Pt macrodisk electrode [13] and will not be extensively discussed here. It is believed that HCl is in its dissociated form of [HCl_2_]^−^ and H^+^ when dissolved in RTILs [13].

(1) $$2{\text{HCl}}_{gas}⇌{\text{H}}^{+}+{\left[{\text{HCl}}_{2}\right]}^{-}$$

**Figure 4:**
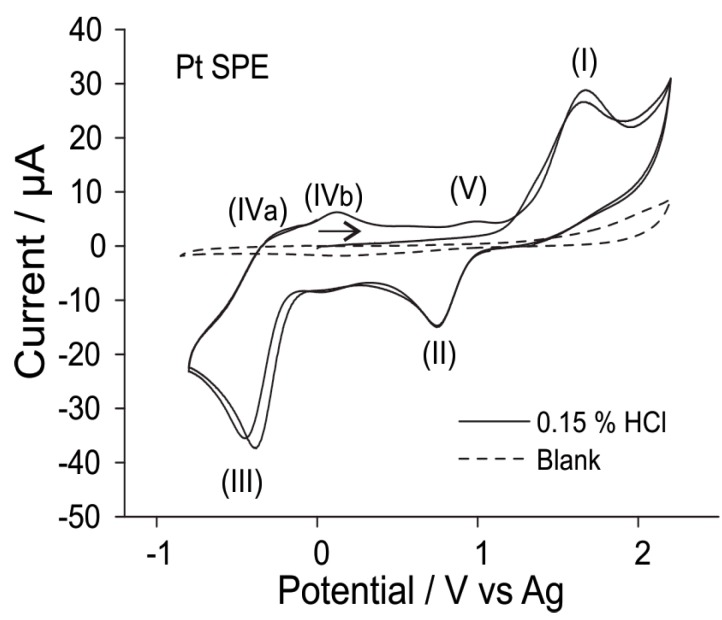
CV (two cycles) for the oxidation of 0.15% hydrogen chloride gas (nitrogen fill) on a Pt SPE in [C_2_mim][NTf_2_] at a scan rate of 0.1 V/s. Dotted line is the response in the absence of hydrogen chloride gas.

Figure 4 shows the electrochemical behaviour of 0.15% hydrogen chloride gas on a Pt SPE scanned from 0 to 2.2 V to −0.9 V and back to 0 V for two CV cycles. On the first cycle, only one oxidation peak (peak (I), at *ca.* 1.65 V) is observed within the available potential window before solvent breakdown. This likely corresponds to the two-electron oxidation of [HCl_2_]^−^ to form H^+^ (solvated by the ionic liquid) and chlorine (Cl_2_) gas. [13] On the reverse sweep, two reduction peaks are observed, corresponding to the reduction of the two electrogenerated products: Cl_2_ (peak (II), at *ca.* 0.78 V) and the reduction of solvated protons (peak III, at *ca.* −0.38 V). Both of these processes have corresponding oxidation peaks: the oxidation of hydrogen (peaks IVa and IVb, two competing processes) [13] and the oxidation of chloride (peak (V), which is present only on the second CV cycle). It is noted that the reduction peak (III) is also present when first scanned in the negative direction (*i.e.*, without first scanning over peak (I)), supporting the above hypothesis (Equation (1)) that solvated protons are present when HCl is dissolved in the RTIL.

**Figure 5:**
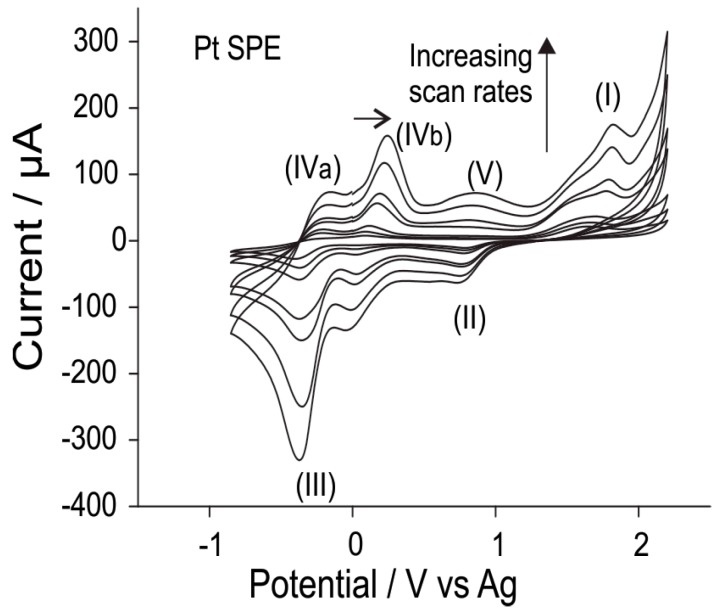
CV (second scan) for the oxidation of 0.15% hydrogen chloride gas (nitrogen fill) on a Pt SPE in [C_2_mim][NTf_2_] at varying scan rates (0.05–3 V/s).

Figure 5 shows CV scans for the oxidation of 0.15% hydrogen chloride gas at varying scan rates between 0.05 to 3 V/s when scanned from 0 to 2.2 V to −0.9 V and back to 0 V (for clarity, only the second scan is shown). It is evident that the peak currents for all processes increase with increasing scan rates. Plots of peak current *vs.* square root of scan rate for peaks (I), (II) and (IVa) and (IVb) are linear, with *R*^2^ values > 0.99, suggesting that the electrochemical processes are most likely diffusion controlled. For the proton reduction peak (peak III), there is also a pre-peak that is especially evident at higher scan rates, which has previously been ascribed to a competing reaction for H^+^ reduction [13,19]. Plots of current *vs.* square root of scan rate for the proton reduction peak (peak III) and the pre-peak (at *ca.* 0 V) are approximately linear (*R*^2^ ~ 0.98–0.99), suggesting that they are also diffusion controlled, but the currents may be slightly influenced by the presence of other peaks.

#### 3.2.2. Analytical Utility of Hydrogen Chloride Gas on Pt SPEs

**Figure 6:**
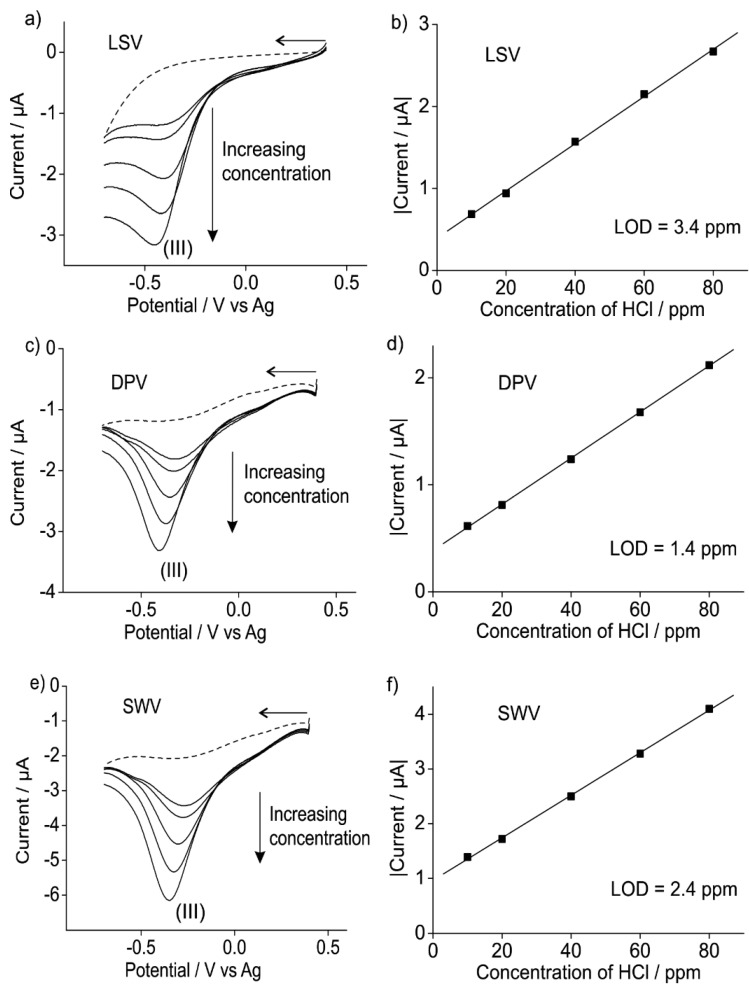
(**a**) LSV, (**c**) DPV and (**e**) SWV for the proton reduction peak at different concentrations of HCl (10, 20, 40, 60 and 80 ppm in the gas phase) on a Pt SPE in [C_2_mim][NTf_2_]. Dashed line is the response in the absence of hydrogen chloride gas. The corresponding calibration plots obtained for (**b**) LSV, (**d**) DPV and (**f**) SWV using the peak currents from (**a**,**c**,**e**) are also shown.

Since the electrochemical mechanism for HCl gas on a Pt SPE was similar to that on a Pt macrodisk [13] electrode, the current *vs.* concentration response for hydrogen chloride gas was studied on Pt SPEs. The proton reduction peak (III) was used as the analytical signal since the oxidation peak (I) is complicated by the appearance of peak (V) on the second scan which adds to the current. Figure 6 shows the results from LSV (6a), DPV (6c) and SWV (6e) experiments for five different concentrations of hydrogen chloride on a Pt SPE for the reduction of H^+^ (peak III). It can be seen that as the concentration increases, the current increases for all three electrochemical techniques. Figure 6b,d,f shows the calibration plots obtained for three techniques and it can be seen that excellent linearity is observed in all plots (*R*^2^ > 0.99). LODs of 3.4, 1.4 and 2.4 ppm with sensitivities of 2.9 × 10^−8^, 2.1 × 10^−8^ and 3.9 × 10^−8^ A/ppm were obtained for LSV, DPV and SWV, respectively. The LODs were calculated using three times the standard deviation (3σ) of the slope of the calibration line. This is comparable to LODs of 2.5, 3.7 and 3.7 ppm for LSV, DPV and SWV, respectively, on a Pt microelectrode (see Figure S2 in the supporting information). Again, the voltammetry on the microelectrode was very noisy due to the much lower currents measured. The LODs obtained are below the OSHA PEL limits of 5 ppm for HCl and suggest that cheap Pt SPEs can be used in conjunction with RTILs for the electrochemical detection of low concentrations of hydrogen chloride.

## 4. Conclusions

The electrochemical behaviour of methylamine and hydrogen chloride gases was investigated on Pt SPEs in an ionic liquid. The mechanism of both gases on Pt SPEs was very similar to that on microelectrodes, *i.e.*, no unusual behaviour on the SPEs was seen, which suggests that the materials in the paste/polymeric binder of SPE_S_ do not react with the toxic gases in a detrimental way (at least on the timescale of the electrochemical experiments). The low LODs obtained (3.2, 3.0 and 3.6 ppm for MA and 2.5, 3.7 and 3.7 ppm for HCl from LSV, DPV and SWV, respectively) suggest that the SPE/RTIL platform can be used to detect these highly toxic gases at low levels in real environments.

## Supplementary Files

Supplementary File 1
