# Supplementary material for: Sensors for Highly Toxic Gases: Methylamine and Hydrogen Chloride Detection at Low Concentrations in an Ionic Liquid on Pt Screen Printed Electrodes
Source: Sensors (Basel). 2015 Oct 22;15(10):26866–76. doi: 10.3390/s151026866 (PMC4634386; doi:10.3390/s151026866)
Supplement: Supplementary File 1 [file sensors-15-26866-s001.pdf]

OPEN ACCESS

*sensors*

**ISSN 1424-8220**

www.mdpi.com/journal/sensors

*Supplementary Information*

# Sensors for Highly Toxic Gases: Methylamine and Hydrogen Chloride Detection at Low Concentrations in an Ionic Liquid on Pt Screen Printed Electrodes. *Sensors* 2015, *15*, 26866-26876

**Krishnan Murugappan † and Debbie S. Silvester ***

Nanochemistry Research Institute, Department of Chemistry, Curtin University, GPO Box U1987, Perth 6845, WA, Australia; E-Mail: d.silvester-dean@curtin.edu.au

† Present address: Department of Materials, Parks Rd, Oxford OX1-3PH, UK.

* Author to whom correspondence should be addressed; E-Mail: d.silvester-dean@curtin.edu.au; Tel.: +61-8-9266-7148; Fax: +61-8-9266-2300.

---

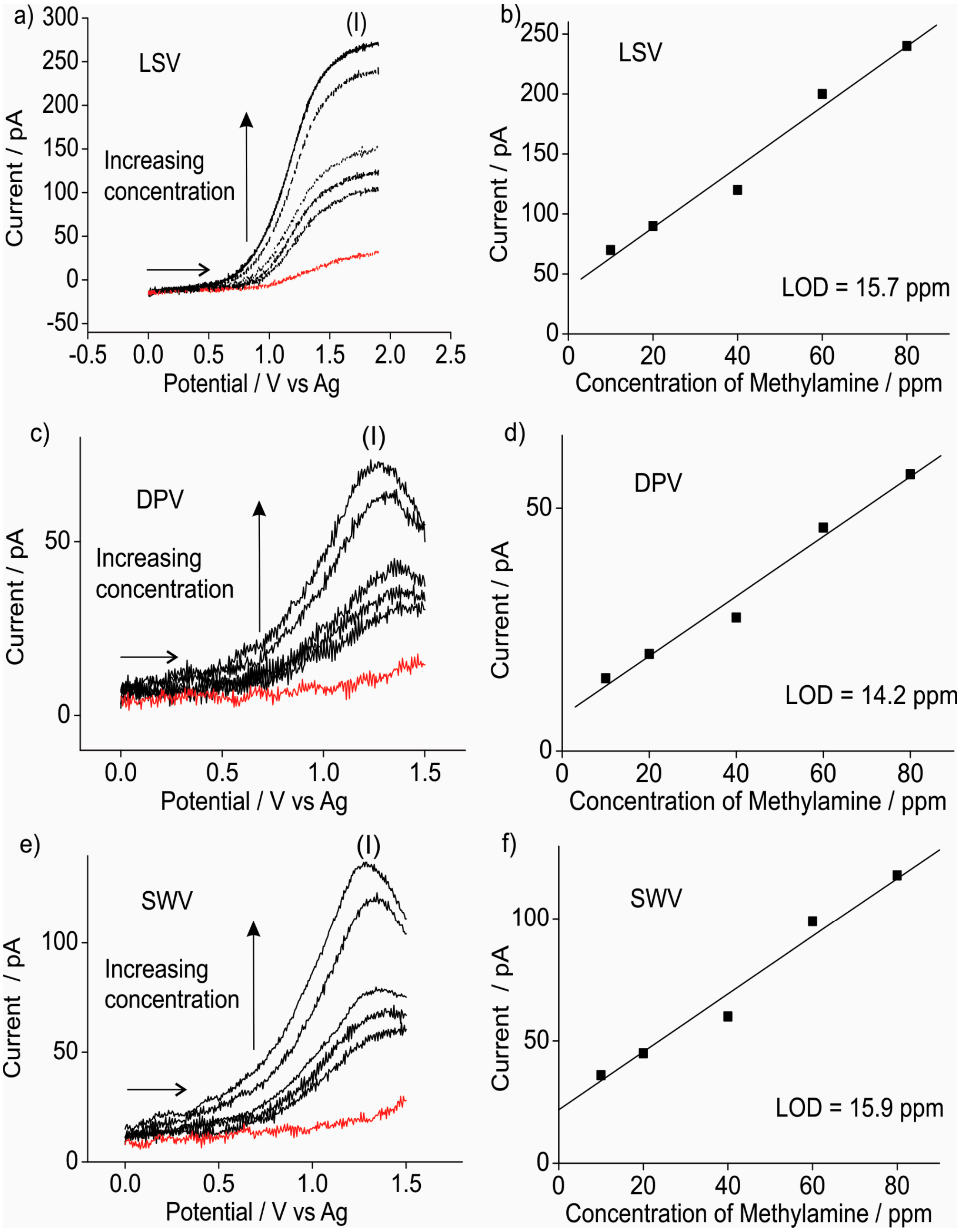


**Figure S1.** (**a**) LSV, (**c**) DPV and (**e**) SWV for the oxidation of methylamine gas (10, 20, 40, 60 and 80 ppm in the gas phase) on a Pt microelectrode (radius 8.3 μm) in $[C_2mim][NTf_2]$. Dashed line is the response in the absence of methylamine gas. Calibration plot obtained for (**b**) LSV, (**d**) DPV and (**f**) SWV using the peak currents from (**a**,**c**,**e**).

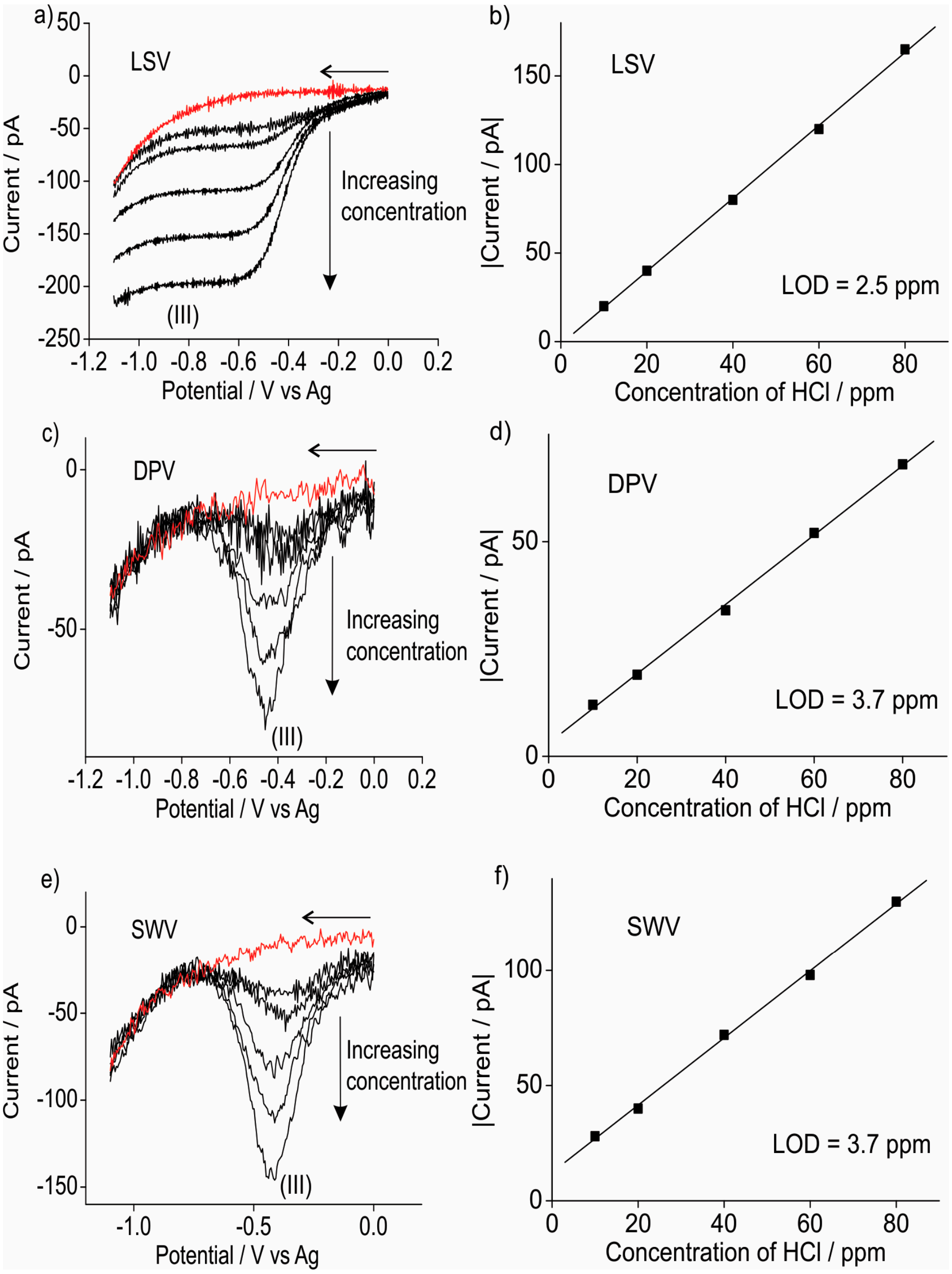


**Figure S2.** (**a**) LSV, (**c**) DPV and (**e**) SWV for the reduction of protons (10, 20, 40, 60 and 80 ppm in the gas phase) on a Pt microelectrode (radius 8.3 μm) in [C2mim][NTf2]. Dashed line is the response in the absence of hydrogen chloride gas. Calibration plot obtained for (**b**) LSV, (**d**) DPV and (**f**) SWV using the peak currents from (**a**,**c**,**e**).

© 2015 by the authors; licensee MDPI, Basel, Switzerland. This article is an open access article distributed under the terms and conditions of the Creative Commons Attribution license (http://creativecommons.org/licenses/by/4.0/).
